# A Living Cell Repository of the Cranio-/Orofacial Region to Advance Research and Promote Personalized Medicine

**DOI:** 10.3389/fcell.2021.682944

**Published:** 2021-06-10

**Authors:** Ludovica Parisi, Patrick O. Knapp, Eleftheria Girousi, Silvia Rihs, Giorgio C. La Scala, Isabelle Schnyder, Alexandra Stähli, Anton Sculean, Dieter D. Bosshardt, Christos Katsaros, Martin Degen

**Affiliations:** ^1^Laboratory for Oral Molecular Biology, Dental Research Center, Department of Orthodontics and Dentofacial Orthopedics, University of Bern, Bern, Switzerland; ^2^Division of Pediatric Surgery, Department of Pediatrics, University Hospital of Geneva, Geneva, Switzerland; ^3^University Clinic for Pediatric Surgery, Bern University Hospital, Bern, Switzerland; ^4^Department of Periodontology, School of Dental Medicine, University of Bern, Bern, Switzerland; ^5^Robert K. Schenk Laboratory of Oral Histology, Dental Research Center, University of Bern, Bern, Switzerland

**Keywords:** patient-derived cells, craniofacial anomalies, cell bank, quality control, rare diseases, personalized medicine

## Abstract

The prevalence of congenital anomalies in newborns is estimated to be as high as 6%, many of which involving the cranio-/orofacial region. Such malformations, including several syndromes, are usually identified prenatally, at birth, or rarely later in life. The lack of clinically relevant human cell models of these often very rare conditions, the societal pressure to avoid the use of animal models and the fact that the biological mechanisms between rodents and human are not necessarily identical, makes studying cranio-/orofacial anomalies challenging. To overcome these limitations, we are developing a living cell repository of healthy and diseased cells derived from the cranio-/orofacial region. Ultimately, we aim to make patient-derived cells, which retain the molecular and genetic characteristics of the original anomaly or disease *in vitro*, available for the scientific community. We report our efforts in establishing a human living cell bank derived from the cranio-/orofacial region of otherwise discarded tissue samples, detail our strategy, processes and quality checks. Such specific cell models have a great potential for discovery and translational research and might lead to a better understanding and management of craniofacial anomalies for the benefit of all affected individuals.

## Introduction

Craniofacial development is a complex process that requires orchestrated interactions of multiple cell types derived from all germ layers (Kindberg and Bush, 2019). Among them, the neural crest cells (NCCs) are the most critical population. NCCs arise at the neural plate border, undergo an epithelial-mesenchymal transition (EMT), and then are guided to the periphery where they form the structures of the head and the face (Hall, 2000; Cordero et al., 2011; Green et al., 2015; Siismets and Hatch, 2020). All these intricate steps rely on precise spatio-temporally regulated networks of signaling cues, which, due to their complexity, are error prone, and may result in a diversity of craniofacial anomalies (CFAs) (Gorlin et al., 1990). In fact, it is estimated that CFAs occur in approximately 0.2% of all newborns (Shaw, 2004). Some, like cleft lip/palate (CLP), are among the most common congenital abnormalities affecting around 1 in 750 newborns (Kousa and Schutte, 2016), while others, such as Goldenhar syndrome (GH) are rarer with an estimated prevalence of 1 in 3000–5000 births (Bogusiak et al., 2017). CFAs can appear in isolation, as part of a syndrome with defects due to mutations in single genes or chromosomal abnormalities, or in combination with other defects without an identified genetic background.

CFA-affected children usually require surgery in order to repair the most debilitating defects, which is routinely followed by personalized treatment throughout childhood and adolescence. Although the surgical procedures have been optimized during the last decades, a number of common complications in CFA-affected children may still persist even after surgery, such as difficulties in feeding, speech and hearing, impaired dentition and wound healing complications (van Beurden et al., 2005; Aizenbud et al., 2011; Hortis-Dzierzbicka et al., 2012). These problems might require additional surgeries, which can be challenging as often not enough tissue is available to correct the anomaly (i.e., alveolar bone defects in CLP patients) (Zhang and Yelick, 2018). In addition, CFAs are still associated with an increased mortality as well as with a negative impact on a child’s self-perception and social integration due to esthetical concerns. Consequently, CFAs represent a high economic, social, and psychological burden for affected individuals, their families, and the society as a whole. Therefore, there is an urgent need for a better understanding of CFAs, which could allow to optimize treatment options tailoring it to the specific patient needs.

Discovery molecular and cellular research on human CFAs is challenging due to the lack of proper models. At the moment knowledge is mostly based on animal studies. Fortunately, craniofacial development is a highly conserved process across many species (Hu et al., 2003) and mice and humans share the majority of protein-coding genes (Yue et al., 2014). However, certain discrepancies exist between humans and animals in regard to facial morphogenesis. For instance, disruption of genes in mice, which have been linked to CLP in humans, often do not perturb the formation of the primary palate and the lip in the animal and hence, produce only a cleft palate (CP) phenotype (Gritli-Linde, 2008; Van Otterloo et al., 2016). As not every finding in animal models can be transferred to humans, and considering the strong urge from the society to minimize animal experimentation by using other studies modalities, CFA patient-derived cells might represent an alternative *in vitro* study tool. However, working with primary cells is often experienced as cumbersome, since they require an established isolation protocol, unique growth media, more care in general, and they exhibit a limited lifespan. The use of postnatal tissues to study CFAs that arise early in development also comes with challenges and assumptions regarding the retention of the original tissue characteristics. However, increasing evidence shows the persistence of various cellular properties postnatally in tissues as well as in patient-derived cells (Beyeler et al., 2014; Hixon et al., 2017; Degen et al., 2018, 2020). In this regard, it is also noteworthy to mention that often it is very ambitious and difficult to have access to proper healthy control tissue and/or cells, which ideally should be age- and anatomically matched to the studied condition (Degen et al., 2020).

To facilitate the use of patient-derived cells as clinically relevant cell models to study CFAs, we have initiated a project to establish a living cell repository of the cranio-/orofacial region starting from routinely discarded tissue biopsies. To this purpose, we collaborate with the Pediatric Surgery Division, Children’s Hospital, Bern, which is responsible for the surgical treatment of CLP-affected infants. In CLP patients, the cleft lip is closed at the age of about 4 months, after removing a small piece of superfluous lip tissue reaching into the cleft. We collect this excessive CLP lip tissue as well as healthy pediatric tissue remnants, obtained from the surgical management of minor injuries (i.e., lip lacerations) often requiring debridement. Similarly, numerous clinical samples are obtained during routine dental treatments, which take place on a daily basis at the Dental School, University of Bern, to which our laboratory is affiliated. Such samples include small tissue biopsies from the gingiva, the periodontal ligament (PDL) and the dental pulp from extracted healthy wisdom teeth, and the hard/soft palate. All these excised tissues are usually discarded since most of these conditions do not require any further diagnostic analyses. Such tissue remnants serve as optimal and easily accessible sources for the isolation of primary cells and for the establishment of a high-quality and readily available cell repository of the cranio-/orofacial region for discovery and translational research.

The aim of this report is to present our proof-of-concept study in creating a robust and high-quality cell bank and in exploiting its full potential for translational applications. Such a unique effort holds great promises in fostering scientific collaborations in the field of cranio-/orofacial development, thereby advancing and promoting personalized precision medicine for unmet medical needs.

## Materials and Methods

### Cell Isolation and Culture

Tissue biopsies were collected in 50 ml conical tubes containing ≈ 25 ml of Dulbecco’s modified Eagle’s medium (DMEM) (Gibco/Life Technologies; Thermo Fisher Scientific, Lucerne, Switzerland) containing 10% fetal calf serum (FCS) (Sigma-Aldrich, St. Louis, MO, United States), 1× Pen/Strep (PS) solution (Gibco), and 1× Amphotericin B (AmphB) (Gibco) [=Collection Medium (CM)]. Samples were transferred to the research laboratory within 2 h (average time) after biopsy collection for further processing. In rare case, when urgent unplanned surgeries/treatments occurred on weekends or during the night, samples were stored overnight at 4°C. We did not notice any negative impact on cell isolation from such samples. Under sterile conditions, the CM was aspirated, and the tissues washed for an additional 5 min in CM. Using sterile tweezers and scalpels, the specimens were minced into small pieces (<1 mm^3^), which were then transferred into six-well plates containing 800 μl of CM and placed in a humidified incubator at 37°C/5% CO_2_. Two pieces were incubated per well of a six-well plate. The explant cultures were carefully washed with CM and re-fed with fresh medium every other day. Cellular outgrowths were daily observed under a light microscope and the cell types growing out of each tissue piece annotated (e.g., fibroblasts only, keratinocytes only, mixed populations). Cells were allowed to grow out to colonies of approximately 0.4 cm diameter before subculturing (passage 1) them into p60 or p100 culture dishes using trypsin/EDTA (TE) solution (Gibco). Pure fibroblast outgrowths were trypsinized using 0.05% TE and pure keratinocyte outgrowths using 0.25% TE. For mixed populations a differential TE approach was used: fibroblasts were detached first, followed by the dissociation of the keratinocytes. Keratinocytes were cultured in Keratinocyte serum-free medium (KSFM) (Gibco/Life Technologies), supplemented with 25 μg/ml bovine pituitary extract, 0.2 ng/ml epidermal growth factor and CaCl_2_ to a final Ca^2+^ concentration of 0.4 mM and 1× PS (Gibco), as previously described (Degen et al., 2012). Fibroblasts were cultured in complete DMEM (Gibco) supplemented with 10% FCS and 1× PS. Cells were expanded in p100 culture dishes, quality-checked, and frozen at passage 2 (2 × 10^5^–1 × 10^6^ keratinocytes/vial and 1 × 10^5^–5 × 10^5^ fibroblasts/vial) in 2× freezing medium (DMEM, 20% FCS, 25 mM HEPES, 20% DMSO) diluted 1:1 in their respective culture medium and stored in liquid nitrogen. For all experiments cells at passage 3 have been used.

For mycoplasma detection, cells were grown for at least 5 days in their specific growth media in the absence of any antibiotics. Afterward cells were fixed in 4% paraformaldehyde (PFA) and the nuclei stained with DAPI using the Vectashield Mounting Medium containing DAPI (Vector Laboratories, Burlingame, CA, United States). In parallel, conditioned medium was collected from the cells, which was used as template for a PCR-based mycoplasma detection analysis as described (Praetorius, 2015).

Bone marrow-derived Mesenchymal Stem Cells (MSCs) (ScienCell, Carlsbad, CA, United States) were cultured in MSC medium (ScienCell).

### RNA Extraction, cDNA Synthesis, and Quantitative Real-Time PCR (qPCR) Analysis

Total RNA was isolated using the innuPREP RNA Mini kit (Analytik Jena AG, Jena, Germany) according to the standard protocol for eukaryotic cells. RNA concentration was subsequently measured with a NanoDrop 2000c (Thermo Fisher Scientific) and stored at −80°C until use.

A 500 ng of total RNA were used as a template to synthetize cDNA using the M-MLV Reverse Transcriptase (Promega, Dübendorf, Switzerland) and Oligo(dT)_15_ Primer (Promega). Gene expression was detected by quantitative real-time polymerase chain reaction (qPCR). qPCR was performed using a GoTaq^®^ qPCR Master Mix (Promega) on a QuantStudio 3 instrument (Applied Biosystems, Thermo Fisher Scientific). Data analysis was performed using the dC_*T*_ method when absolute mRNA normalized to *GAPDH* levels were reported, or by ddC_*T*_ method when absolute mRNA normalized to *GAPDH* were further referenced to a control sample set to 1.

Sequences of the qPCR primers (Supplementary Table 1) were either taken from the PrimerBank database^1^ or from the NCBI primer designing tool^2^. All qPCR primer pairs were tested for specificity and efficiency using cDNA standard curves.

### Immunoblotting

Whole cell extracts were prepared in 1× RIPA buffer (10 mM Tris–HCl pH 8.0, 1 mM EDTA, 0.1% sodium deoxycholate, 0.1% SDS, 1% NP40, 140 mM NaCl) supplemented with cOmplete Mini^TM^ Protease Inhibitor cocktail and PhosSTOP EASYpack (both from Sigma-Aldrich). Protein concentrations were measured using the BCA Protein Assay kit (Pierce, Thermo Fisher Scientific) following the manufacturer’s recommendation. 10 μg of total protein were diluted in sample loading buffer (62.6 mM Tris–HCl pH 6.8, 2% SDS, 10% glycerol, 0.01% bromophenol blue) containing 100 mM dithiothreitol (DTT) (Sigma-Aldrich), boiled for 5 min at 95°C and separated under reducing conditions by SDS-PAGE. Proteins were subsequently blotted onto nitrocellulose membranes (Sigma-Aldrich). Membranes were stained with 0.1% amido black solution (Merck, Burlington, MA, United States) to control for equal protein loading and blotting efficiency. Afterward, membranes were washed in Tris-buffered saline (TBS-Tween) (pH 7.4) containing 0.05% Tween-20, blocked in 3.5% skim milk powder, incubated over-night with primary antibody at 4°C, washed three times in TBS-Tween buffer and incubated with horseradish peroxidase-conjugated anti mouse or rabbit IgG. Blots were developed using the SuperSignal West Dura kit (Pierce, Thermo Fisher Scientific). Data were acquired by scanning membranes with an Imager Chemi Premium Instrument (VWR, Darmstadt, Germany). Primary antibodies used are reported in Supplementary Table 2.

Some immunoblots were analyzed densitometrically using the ImageJ software, version 1.53g (NIH; Bethesda, MA, United States). Briefly, the intensity of each protein band was normalized to the β-actin or vinculin band intensity of the same extract in the same experiment.

### Lifespan Analysis

For replicative lifespan analysis, cells were plated at a density of 10^4/^p60 dish for keratinocytes and 10^5/^p100 for fibroblasts in their standard growth media. Cells were re-fed every other day and subcultured when reaching 80% of confluency. The day of subculturing, cells were counted using a Countess 2 automated cell counter (Thermo Fisher Scientific) and re-seeded at the same initial cell density. Population doublings (PD) were calculated as log*2* (# cells at the time for subculturing/# cells plated), while cumulative PDs were plotted against the total days in culture.

### Immunofluorescence

For stainings, cells were grown in 35 mm dishes containing four separate wells (Greiner Bio-One, Frickenhausen, Germany). Fixation occurred in 4% paraformaldehyde at room temperature (RT) for 20 min when keratinocytes formed colonies of 8–10 cells and fibroblasts reached 50% of confluency. Afterward, cells were rinsed 3 times in Phosphate Buffer Saline (PBS), permeabilized with 0.1% Triton-X-100 for 5 min, blocked in 3% Bovine Serum Albumin (BSA) (Sigma-Aldrich) for 15 min and incubated with primary antibody at RT for 2 h. Cultures were washed three more times in PBS and incubated with a fluorescent-labeled secondary antibody (Molecular Probes, Thermo Fisher Scientific) with or without tetramethylrhodamine (TRITC)-phalloidin (Sigma-Aldrich) for 1 h protected from the light, washed with PBS and ddH_2_O, and coverslip mounted with Vectashield Mounting Medium containing DAPI (Vector Laboratories). All the samples were examined under an Olympus BX-51 phase/fluorescence microscope (Olympus Life Science Solution Tokyo, Japan) equipped with a xenon lamp (X-Cite, series 120PC Q, Lumen Dynamics, Mississauga, Canada), and fluorescence filters U-MWIBA3 for Alexa Fluor 488, U-MWIGA3 for Alexa Fluor 568 and TRITC, and U-MNUA2 for DAPI (Olympus Life Science Solutions). Images were captured by a ProgRes CT3 camera with ProgRes Capture Pro software (Jenoptik, Jena, Germany). Primary antibodies used are reported in Supplementary Table 2.

### Histology

In order to prepare formalin-fixed and paraffin-embedded (FFPE) blocks, tissue specimens were fixed in 10% formalin, which is roughly equivalent to 4% formaldehyde, for 48 h at RT. Samples were then dehydrated with increasing alcohol concentrations, followed by changes in xylene, before embedding in paraffin. Five to six-μm sections were finally cut on a Reichert-Jung microtome (Leica Microsystems, Wetzlar, Germany), deparaffinized and stained with hematoxylin-eosin (H&E). Alternatively, after fixation, samples were embedded in acrylic resin (LR White resin, Sigma-Aldrich), cut into 1 μm sections with a diamond knife and stained with Toluidine Blue.

All immunohistochemical (IHC) staining reactions were performed by automated staining using a BOND RX autostainer (Leica Microsystems). Sections were deparaffinized and antigen was retrieved using 1mM Tris solution (pH 9.0) for 30 min at 95°C. Sections were stained with primary antibodies (Supplementary Table 2), followed with secondary antibodies, and specific binding of primary antibodies was visualized using a polymer-based visualizing system with horseradish peroxidase as the enzyme and 3,3-diaminobenzidine (DAB) as a brown chromogen (Leica Microsystems). Finally, the samples were counterstained with hematoxylin and mounted with Aquatex (Merck, Burlington, MA, United States). To reveal cartilage, sections from the GH patient were stained with Safranin-O. Briefly, sections were deparaffinized and stained with 0.1% Safranin-O solution (Sigma-Aldrich) for 5 min at RT, followed by rinses with ddH_2_O and nuclei counterstaining using hematoxylin. After extensive washes under running tap water, samples were mounted using resinous medium.

All samples were examined under an Axio Imager M2 microscope (Carl Zeiss, Oberkochen, Germany) and images taken using a digital AxioCam MRc camera (Carl Zeiss).

### 3D-Skin Models

To generate 3D-skin models using patient-derived keratinocytes, the protocol from CELLnTEC (CELLnTEC advanced cells systems AG, Bern, Switzerland) was used. Briefly, cells were seeded in 400 μl KSFM into polycarbonate inserts (0.4 μm pore size, 12 mm diameter, Nunc, Thermo Fisher Scientific) placed in 60 mm tissue culture dishes, immediately followed by the addition of 11 ml of KSFM outside the inserts. Confluent monolayer formation was confirmed with the staining kit (CnT-ST-100, CELLnTEC). If confluent, keratinocyte differentiation was induced in parallel cultures by the replacement of KSFM with 3D Barrier Medium (CnT-PR-3D, CELLnTEC) inside and outside of the insert (equal medium level) overnight. The insert was then lifted to the air-liquid interface by carefully removing the medium inside the insert and replenishing the outside medium with fresh 3D Barrier Medium up to the level of the membrane (approximately 3.2 ml for a p60 culture dish). Keratinocytes were cultured for 15 days at 37°C and 5% CO_2_ with medium change every other day. For full-thickness skin models, fibroblasts were cultured without any antibiotics and seeded into polyethylene terephthalate (PET) inserts at 5 × 10^4^ cells per insert and grown for 10 days before adding keratinocytes on top in a fully defined co-culture medium for culture at the air liquid-interface (CnT-PR-FTAL, CELLnTEC). Three days after seeding the keratinocytes on top of the dermal layer, the models were air-lifted and grown for further 12 days with medium changes three times per week.

To analyze the skin models, 3D-cultures were fixed overnight at 4°C in 4% PFA. Membranes were cut out of the inserts, placed between two biopsy pads in embedding cassettes, and stored in 0.1 M sodium cacodylate buffer at 4°C before proceeding for paraffin embedding and sectioning on a Reichert-Jung microtome (Leica Microsystems). Slides containing 5 μm thick paraffin sections were deparaffinized and rehydrated through xylene, ethanol, and ddH_2_O, stained with H&E or primary antibodies as described in section Histology. Primary antibodies used are reported in Supplementary Table 2.

### MTT-Assay

To evaluate the cytotoxic effects of a commercially available oral rinse (OR) on gingival fibroblasts and keratinocytes, an MTT (3-(4,5-dimethylthiazol-2-yl)-2,5-diphenyltetrazolium bromide) assay was performed (Sigma-Aldrich). Briefly, cells were seeded in triplicates at a density of 300 cells/mm^3^ in 96-well plates. 24 h after seeding, they were rinsed twice in PBS and exposed to different concentrations of the OR for 2 min. Subsequently, cells were incubated with MTT solution at a final concentration of 0.5 mg/ml for 4 h to allow MTT conversion into formazan in metabolically active cells. After two PBS washes, converted MTT was solubilized with 4N HCl and the absorbance read at 570 nm on an EL808 BioTek microplate reader (BioTek, Winooski, VT, United States).

### *In vitro* Differentiation

For *in vitro* differentiation, MSCs and PDL fibroblasts at a density of 10^5^ cells/well of a six-well plate were seeded in their respective growth medium. 24 h afterward, medium was replaced with osteogenic medium [αMEM (Thermo Fisher Scientific) supplemented with 0.05 mg/ml L-Ascorbic acid, 0.01 M β-Glycerol Phosphate, and 0.1 mM Dexamethasone (all from Sigma-Aldrich)] for 21 days with medium change every third day. At the end of the culturing time, cells were fixed in 4% PFA for 20 min at RT, rinsed with ddH_2_O and finally incubated with Alizarin Red S solution [68.45 mg Alizarin Red S (Sigma-Aldrich) in 5 ml ddH_2_O, pH 4.1–4.5] for 45 min at 4°C protected from light. After extensive washing in ddH_2_O, samples were air-dried and analyzed with a light microscope.

### Statistical Analysis

Experiments were performed at least three times in multiple replicates. Data were analyzed using Prism 7 (GraphPad; La Jolla, CA, United States). Data are represented as means ± standard deviation (SD). Multiple comparisons were performed using one-way analysis of variance (ANOVA) with Tukey’s *post hoc* test. Values of *p* ≤ 0.05 were considered significant.

## Results

### Establishment of a Solid and Reproducible Method for Keratinocyte and Fibroblast Isolations From Routinely Discarded Tissues of the Cranio-/Orofacial Region

**FIGURE 1:**
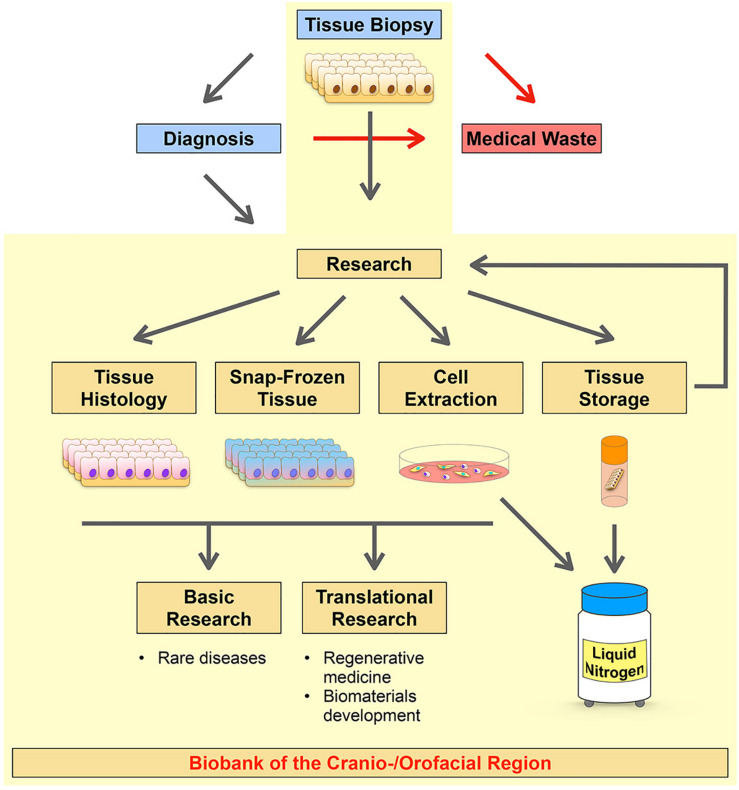
Schematic diagram summarizing the strategy of our CFA biobank. The Pediatric Surgery Division, Children’s Hospital, Bern, and the Dental School, University of Bern are responsible for tissue excisions and, if required, for further diagnostic analyses of the biopsies (blue boxes). Usually, tissues from the cranio-/orofacial region are discarded after their initial excision (no diagnosis required) or after their diagnostic analysis (medical waste, red box). The research laboratory (Laboratory for Oral Molecular Biology, University of Bern) collects such specimens and processes them in order to create a living cell repository of patient-derived cells (Cell Extraction). After isolation of the cells, if the tissue size allows it, the remaining tissue remnants are (1) stored in freezing medium in the liquid nitrogen tank for future processing and new cell extractions (Tissue Storage), (2) formalin-fixed and paraffin-embedded (FFPE) for histological analyses (Tissue Histology), and (3) snap-frozen for future tissue RNA and/or DNA extractions (Snap-Frozen Tissue). These patient-derived resources can be used for several downstream applications in discovery and translational research projects. Our workflow is highlighted in yellow.

To advance and promote discovery and translational research on CFAs we initiated a long-term project in establishing a living cell repository of the cranio-/orofacial region. The initial aim was to collect routinely discarded tissues made available by the Pediatric Surgery Division, Children’s Hospital, Bern, and the Dental School, University of Bern. While we put our priority on the establishment of a highly robust and reproducible process for the isolation of patient-derived cells, our strategy also allowed us to collect and biobank corresponding tissues for histological analyses and snap frozen tissues for RNA and DNA extractions (Figure 1).

**FIGURE 2:**
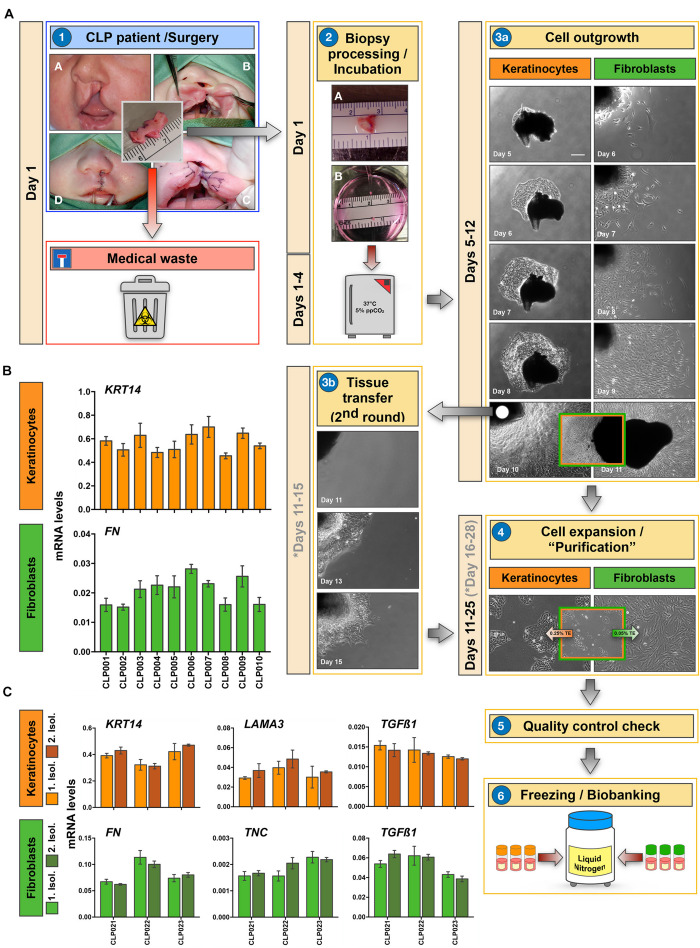
Establishment of a robust and reproducible method for the isolation of keratinocytes and fibroblasts. **(A)** Illustration of the process we follow for the isolation of patient-derived cells from tissue biopsies. A representative example for a CLP biopsy is shown. Briefly, lip tissue from a corrective CLP surgery, which would be routinely discarded (medical waste), is collected in Collection Medium (CM) and transferred to the laboratory (1). After mincing the tissue biopsy, two small tissue fragments of about 0.1 mm^3^ are put in one well of a six-well culture plate containing 800 μl of CM (2). These six-well plates are then incubated at 37°C/5% CO_2_, re-fed every other day, and observed under the microscope for cellular outgrowths (3a). After approximately 10 days, outgrowths are usually ready for subculturing (3a). Cells are trypsinized, expanded in their respective medium, quality checked, and finally frozen (at passage 2) in freezing medium and stored in a liquid nitrogen tank (4–6). Note that in case of mixed keratinocyte/fibroblast cultures, differential trypsinization using 0.05% trypsin-EDTA (TE) solution for detaching fibroblasts, followed by 0.25% TE to dissociate keratinocyte is applied (4). Also note that tissue pieces can be re-used for another round of cell isolation if required (3b). Approximate incubation times (depends on cell type and tissue biopsies) are indicated in days. If a second round of cell isolation is required from an initial tissue piece, the days are indicated in gray. Scale bar: 100 μm. **(B)** qPCR analyses of ten individual non-syndromic CLP cell cultures for the epithelial marker *Keratin14* (*KRT14*) and the mesenchymal marker *Fibronectin* (*FN*). Although cells have been isolated from 10 different tissue donors, keratinocytes (orange) and fibroblasts (green) express similar levels of *KRT14* and *FN*, respectively among the cohort. *n* = 3 (biological replicates). **(C)** Analyses of CLP cells derived from three individual donors (CLP021-CLP023) from two distinct cell isolations to show reproducibility of our cell isolation protocol. In between the first and the second cell isolation (light vs. dark orange for keratinocytes, and light vs. dark green for fibroblasts), the tissue remnants have been stored in freezing medium in the liquid nitrogen tank for at least 3 years. qPCR shows comparable expression of *KRT14*, *Laminin* α3 (*LAMA*3), and *Transforming Growth Factor* β*1* (*TGF*β1) in keratinocytes (orange), and of *FN*, *Tenascin-C* (*TNC*) and *TGF*β1 in fibroblasts (green) derived from two consecutive cell isolations (Isol. 1: Isolation 1; Isol. 2: Isolation 2). *n* = 3 (technical replicates).

The core of our biobank is represented by a collection of patient-derived cells of the cranio-/orofacial region. Our main goal is to specifically collect epithelial cells (keratinocytes) and the corresponding mesenchymal cells (fibroblasts) from the same tissue donor by the explant culture approach (Figure 2A). The opportunity to collect both cell types from the same individual will allow the establishment of relevant 3D-co-culture systems that closely mimic the *in vivo* situation, or the analysis of the preferred individual cell type in line with specific research interests in the future (see section “Our Cell Bank Offers Novel Tools and Models for Personalized Precision Medicine”).

To successfully biobank all materials as described in our strategy, the tissue biopsy we receive should ideally have a minimal size of 0.1 cm^3^. With this specimen size we are able to isolate keratinocytes and fibroblasts, prepare FFPE blocks, snap-freeze the sample, and store some of the tissue biopsy in liquid nitrogen for future use (Figure 1). When we receive tissues <0.1 cm^3^, we prioritize our work and focus on cell isolations and storage of the tissue in freezing medium in the liquid nitrogen.

**FIGURE 3:**
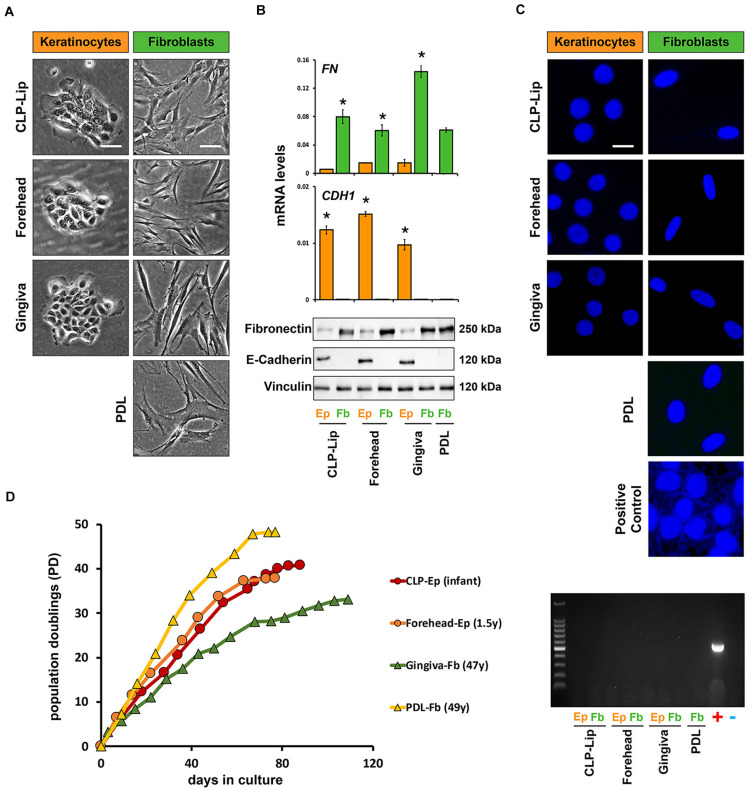
Before biobanking, newly isolated cell cultures undergo a standardized quality control check. **(A)** In a representative set of primary cells (CLP-Lip, Forehead, Gingiva, and PDL) live imaging pictures show typical cell morphologies of the patient-derived keratinocytes (orange) and fibroblasts (green). While keratinocytes form tightly packed and cohesive colonies, fibroblasts appear with an elongated and spindle-like shape. Scale bar: 50 μm. **(B)** The same set of primary cells was used for qPCR (top) and immunoblot analysis (bottom) confirming E-Cadherin (*CDH1*) and fibronectin (*FN*) expression in keratinocytes (orange bars) and fibroblasts (green bars), respectively. Data are expressed as mean ± SD. *n* = 3. **(C)** Mycoplasma contamination of the new cell cultures is tested by DAPI staining of the nuclei and a PCR-based mycoplasma detection analysis. DAPI staining and PCR amplifications are shown to confirm absence of mycoplasma contamination in the same representative set of newly isolated primary cell cultures. Scale bars: 25 μm (Live Imaging keratinocytes), 50 μm (Live Imaging fibroblasts), 10 μm (DAPI). +positive control; –negative control; PDL: periodontal ligament. *p < 0.05 Ep vs. Fb. **(D)** Lifespan analysis of two representative keratinocyte (CLP lip, red, and forehead, orange) and two fibroblast strains (gingiva, green, and PDL, yellow) reveals that all cell cultures tested have a replicative potential of at least 35 population doublings (PD). The age of the tissue donors is indicated in the brackets. Y: year; Ep: epithelial cells; Fb: fibroblasts.

**FIGURE 4:**
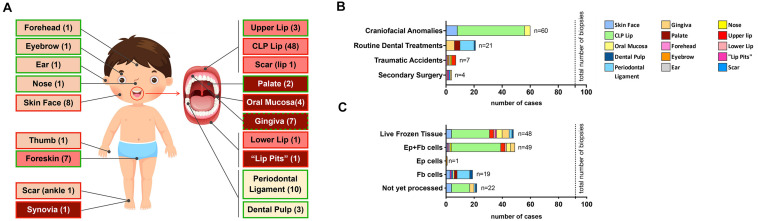
Overall description of our living cranio-/orofacial cell repository. **(A)** Graphical illustration indicating the anatomical origin of stored tissue biopsies (including non-cranio-/orofacial-derived specimens). Light pink indicates skin-derived specimens; pink indicates mucocutaneous junction area-derived specimens; red indicates mucosal-derived specimens; while light yellow indicates tooth-derived specimens. Furthermore, a green border depicts healthy tissue while a red border represents tissue with anomalies. For the gingiva, we have both healthy and diseased tissues (green and red border). The number in the boxes in brackets represents the amount of tissues available. **(B)** Bar chart reporting the source of the specimens that were obtained for the generation of the living cell biobank. Total number of cranio-/orofacial region biopsies: 92 biopsies. **(C)** Bar chart of the outcome after tissue biopsy processing. The color code is shown in the legend. Total number of biopsies: 92.

**FIGURE 5:**
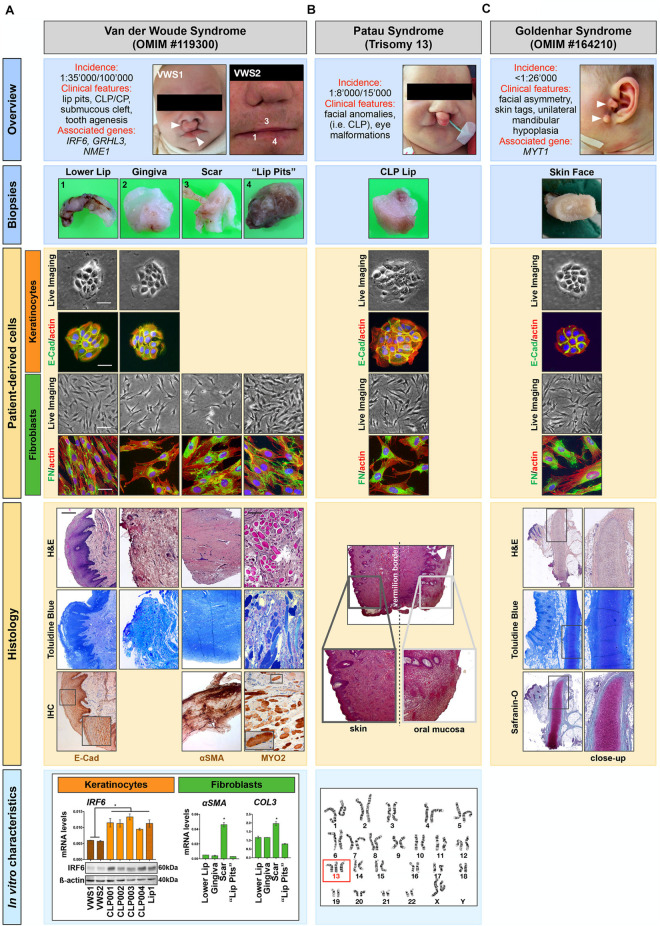
Patient-derived cells can serve as promising research tools for studying rare human diseases. Our cohort of tissue donors encompasses individuals affected by **(A)** Van der Woude syndrome (VWS), **(B)** Patau syndrome, and **(C)** Goldenhar syndrome (GH). *Overview*: A brief description of the rare human diseases is shown as well as the clinical appearance of the tissue donors. Arrowheads indicate typical phenotypes such as bilateral lip pits and orofacial clefts (VWS1, **A**), and pathological skin tags in the GH-affected individual **(C)**. *Biopsies*: High magnification images of the tissue biopsies taken for histological analysis are depicted. Note that the biopsies shown for the VWS were all derived from a secondary surgery of a 19 years old individual (VWS2) and are indicated by numbers (1, 3, 4) in the “Overview” **(A)**. *Patient-derived cells*: Explant cultures allowed the isolation of patient-derived cells of the biopsies shown, which were thoroughly characterized by morphological as well as immunofluorescent analyses. All keratinocytes (orange) show the typical colony-forming morphology (Live Imaging) and are all positive for the epithelial marker E-Cadherin (E-Cad), while fibroblasts (green) present as elongated cells (Live Imaging) positive for the mesenchymal marker Fibronectin (FN). Note that explant cultures from the scar and the “lip pits” of the VWS2 individual **(A)** only resulted in fibroblasts. Scale bars: 25 μm (Live Imaging keratinocytes), 50 μm (Live Imaging fibroblasts), 25 μm (IF). *Histology*: Representative Toluidine Blue and/or H&E stainings of the tissues, with some close-ups are shown. For the VWS2-derived samples additional immunohistochemical analyses (IHC) were performed **(A)**. The expression of E-Cad indicating the skin epithelium was revealed in the lower lip sample. Similarly, the scar tissue revealed excessive staining for α-smooth muscle actin (α-SMA). Note also the abundant amount of striated muscles in the biopsy derived from the VWS2 “lip pits” connective tissue [anti-myosin II (MYOII) immunoreactivity and H&E/Toluidine Blue staining]. In the H&E staining of the Patau syndrome biopsy **(B)** the transition zone between skin and mucosa of the lip can be appreciated (Vermillion border). Finally, Safranin-O staining of the skin tag biopsied from the GH patient identifies the presence of pathological cartilage formation **(C)**. Scale bar: 0.1 mm. *In vitro characteristics*: **(A)** qPCR and immunoblot analyses show that the levels of IRF6 in VWS1 and VWS2 keratinocytes are significantly decreased compared to the levels in 4 non-syndromic [wild-type for the VWS-associated genes *IRF6* (NM_006147.4), *GRHL3* (NM_198173), and *NME1* (NM_198175)] CLP and one healthy lip keratinocyte cell cultures. These results strongly suggest that the VWS2 individual harbors an *IRF6* variant [this has been confirmed for VWS1 by genetic analysis (Degen et al., 2020)] (left). Additionally, fibroblasts isolated from the scar tissue of VWS2 showed higher expression of α*-SMA and Collagen type III (COL3)* mRNA levels compared to fibroblasts isolated from the same donor but from different origins (gingiva, lower lip, and “lip pits,” right). Thus, the original characteristics are retained in the patient-derived cells. Data are expressed as mean ± SD. *n* = 3. * = *p* < 0.05. **(B)** Presence of three chromosomes 13 in the patient affected by the Patau syndrome is shown by karyotype analysis (red box).

**FIGURE 6:**
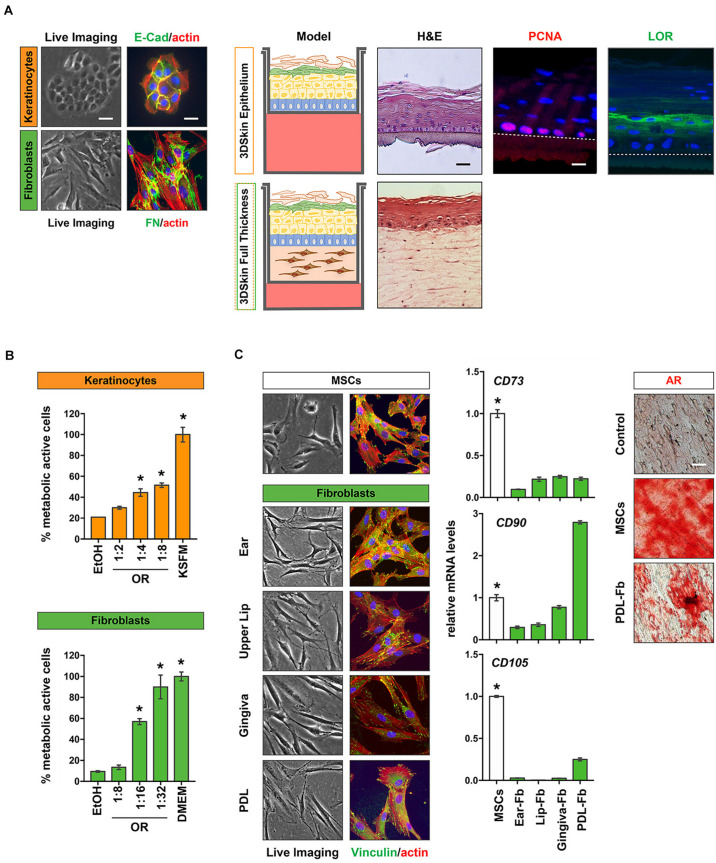
Potential outlook for the use of patient-derived cells from the cranio-/orofacial region. **(A)** CLP patient-derived keratinocytes (orange) and fibroblasts (green) were isolated from CLP patients and characterized by staining for E-Cad (keratinocytes) and FN (fibroblasts). Scale bars: 50 μm (Live Imaging), 25 μm (IF). These cells were used to obtain 3D skin differentiation models (top) that can be stained for H&E, the proliferation marker PCNA (red) and Loricrin (LOR, green). Note that PCNA is only detectable in basal keratinocytes, whereas LOR is present in more superficial keratinocyte layers confirming the *in vivo* situation. Dotted line indicates the membrane. Scale bars: 50 μm (Live Imaging), 25 μm (IF). Alternatively, the isolation of both keratinocytes and fibroblasts from the same tissue donor allows the establishment of full-thickness 3D-skin models, in which CLP keratinocytes are plated on a dermis created by the corresponding fibroblasts that also can be stained (H&E, bottom). *p < 0.05 EtOH vs. oral rinse or KSFM or DMEM. **(B)** Healthy gingival keratinocytes (orange) and fibroblasts (green) were used to determine cytotoxicity of a commercially available oral rinse (OR) at different dilutions after a 2 min exposure by MTT assay. Metabolic active cells are plotted as percentage of the initial cell population. **(C)** Cranio-/orofacial derived fibroblasts share similar characteristics with MSCs and may have a potential in personalized regenerative therapeutic approaches. Like MSCs, fibroblasts have a spindle-like morphology and are plastic-adherent, as revealed by live imaging and vinculin staining (green). Furthermore, qPCR analyses revealed that our patient-derived fibroblasts also express the typical MSC markers *CD73*, *CD90*, and *CD105*. *p < 0.05 MSC vs. Fbs. Alizarin Red S (AR) staining indicates the potential of PDL fibroblasts to be differentiated into bone forming cells. Scale bar: 20 μm. Data are expressed as mean ± SD. *n* = 3.

Tissue samples are collected in sterile 50 ml tubes containing 25 ml of Collection Medium (CM). Within less than 1 h after biopsy, tissue samples are processed (Figure 2A-2). For cell isolations we apply the explant culture technique, which is extensively described in section “Cell Isolation and Culture.” First cells usually emerge within 4 days at 37°C, and colonies are ready for subculturing after approximately 10 days in culture (Figure 2A-3a). Pure cells are expanded (Figure 2A-4), checked for quality (Figure 2A-5, also see section “Cell Isolation and Culture”) and finally frozen (usually 11 cryovials) for long-term storage in liquid nitrogen (Figure 2A-6).

If required, tissue pieces with successful cell outgrowths can be carefully transferred into a new well of a six-well plate containing 800 μl of CM allowing a 2nd round of explant cultures from the same tissue (Figure 2A-3b). This possibility is especially useful when the initial biopsy is rather small. Note that each of the fibroblast and keratinocyte primary cell cultures originating from individual donors represent a mixture of fibroblasts and keratinocytes that grew out of multiple explants.

In our experience, we do get successful cellular outgrowths from approximately 75% of the small tissue pieces. Emergence of the first cells is usually observed 3–4 days after initiation of the explant cultures. Keratinocytes are often growing out of the tissues faster than fibroblasts, which is beneficial as the keratinocytes help to attach the tissue pieces to the culture dish.

In order to minimize the risk of inter-experimental variations during cell isolation, which would hamper any future comparative studies, we follow a highly reproducible workflow. Here, our main rules for cell isolations are detailed. First of all, tissues are all processed in the research laboratory within 1 h after collection. In rare cases, if this is not possible, tissues are stored at 4°C for longer times, and this is properly annotated in the digital databank platform. Secondly, only a team of three well-trained laboratory scientists is in charge of the practical performance of the explant cultures. Thirdly, a strict adherence to Standard Operating Procedures (SOPs) is maintained. SOPs rigorously define the outline of the practical work as well the responsibilities along the cell isolation process, starting from the tissue collection until the final inclusion of the sample into the biobank database. To test whether following these rules was enough to establish a highly robust and reproducible cell isolation process, we tested ten cell strains that have been isolated from ten different non-syndromic, age-matched CLP lip tissue donors for the variability in mRNA levels of some genes. We assumed that patient-derived keratinocytes as well as fibroblasts from the same passage and cultured to the same cell density should display similar levels of the genes *Keratin14* (*KRT14*) and *Fibronectin* (*FN*), respectively. Figure 2B clearly indicates that *KRT14* and *FN* are very evenly expressed within the group of tissue donors tested (CLP001-CLP010 *p* > 0.05). Our biobank strategy also includes the storage of tissue remnants in freezing medium in liquid nitrogen for potential cell re-isolation (secondary isolation). Robustness and reproducibility of our cell isolation protocol should result in identical cells after initial and secondary isolation from the same tissue donor. Therefore, we re-isolated cells from three individual tissue biopsies (CLP021-CLP023) that have been stored in freezing medium in the liquid nitrogen tank for more than 3 years (initial cell isolations: 2016–2017; secondary cell isolations: 2020–2021) according to our established SOP (Figure 2A). Keratinocytes and fibroblasts from secondary isolations were indistinguishable from the initially isolated cells in regard to cell growth and morphology (data not shown). Moreover, we used a qPCR approach to determine gene levels of the two independent isolations. Cells of the same passage (passage 3) and at the same density (60%) were compared. In keratinocytes (orange), *KRT14*, *Laminin*α*3* (*LAMA3*), and *Transforming Growth Factor* β1 (*TGF*β1) levels were indistinguishable in the original (light orange) and the “secondary” cells (dark orange) isolated from the same tissue donor (Figure 2C). Similarly, we did not detect any significant differences in the levels of *FN*, *Tenascin-C* (*TNC*), and *TGF*β1 in the fibroblasts from the primary (light green) vs. secondary (dark green) cell isolations (Figure 2C). The robustness of our explant assay guarantees that variations among individual patient-derived cell strains in regard to gene expression are only minimal as long as cell density, donor age, and anatomical site are comparable. Therefore, whenever a significant difference in gene or protein level is detected, this change might be real and relevant for the condition studied. In summary, we show that we have established a highly robust and reproducible explant culture protocol for the isolation of patient-derived fibroblasts and keratinocytes, which allows comparative studies as well.

### Patient-Derived Cells Have to Successfully Pass a Quality Control Analysis Before Being Biobanked

Problems associated with the establishment of new cell strains, such as misidentification or mycoplasma contamination, are often ignored by the research community and may lead to unreliable research data (Drexler and Uphoff, 2002). Therefore, prior to biobanking, our newly isolated cells are submitted to a standardized quality control procedure (Figure 3).

#### Morphology and Gene Expression

To gain pure fibroblast and keratinocyte cultures, we have to avoid possible contaminations with the counterpart cell type. The morphologies of fibroblasts and keratinocytes allow the simple monitoring of cross-contaminations by brightfield microscopy and, if required, the two cell types are purified by the differential trypsinization approach as described above. Pure keratinocyte cell cultures suitable for biobanking should form regularly shaped and cohesive colonies (Figure 3A, first column), while fibroblast cultures should appear as homogenous monolayers of flat, elongated and spindle-shaped cells (Figure 3A, second column). However, cell purity and identity are not only assessed by visual examinations of their morphologies, but also by qPCR and immunoblots for epithelial and mesenchymal markers (epithelial markers: E-Cadherin, Laminin γ2 and Keratin14; mesenchymal markers: Fibronectin, Vimentin and Tenascin-C). Figure 3B shows that the epithelial cells (Ep, orange bars) only express E-Cadherin, but not Fibronectin, and *vice versa* for the mesenchymal cells (Fb, green bars).

#### Mycoplasma Contamination Detection

Although we strictly follow the rules of good laboratory practice, there is always the risk of mycoplasma contamination when working with primary cell cultures. The original source of tissue serves as the most probable source of mycoplasma in primary cell cultures, but contamination can also occur from exogenous factors (Barile and Rottem, 1993; Rottem et al., 2012). Testing for potential mycoplasma contaminations is a prerequisite for setting up a living cell repository. We routinely test all our newly isolated cell cultures for mycoplasma contamination by a DAPI- and PCR-based detection approach (Praetorius, 2015). Figure 3C shows examples of nuclei stained by DAPI without any signs of mycoplasma contamination in combination with the corresponding PCR-based mycoplasma detection test. A primary cell culture that is found to be contaminated is discarded so that only mycoplasma-negative cells are finally biobanked. So far, only one cell strain derived from a CLP patient was found to be mycoplasma-positive and hence excluded from the biobank.

#### Lifespan Assessment

Primary cell cultures have a finite lifespan. In order to promote them as promising cell models to study human CFAs, the patient-derived cells must possess a replicative potential allowing successful experimental manipulations. To assess this requirement, we regularly perform lifespan analyses with our newly isolated fibroblast and keratinocyte cultures. Figure 3D indicates that our patient-derived cells can be consistently propagated beyond 35 population doublings (PDs). This is substantially shorter than what has been reported for infant foreskin epidermal keratinocyte primary lines such as strain N, but similar to the lifespan of other p63-positive human epithelial cell types (Dabelsteen et al., 2009). It is evident that the replicative lifespan depends on cell types, cell origin, and donor age, which makes such comparisons difficult. Still, we are able to routinely grow our cells for more than 80 days and keep them healthy growing up to passage 11, which makes them highly suitable for standard research activities.

### CFA BIOBANK – State of Our Living Cell Repository

Since April 2016, we received a total of 92 tissue biopsies from the cranio-/orofacial region of 71 different donors (Table 1). Samples can be grossly classified into four distinct groups in respect to their origin: (1) skin-derived specimens (*n* = 12; forehead, eyebrow, ear, nose and facial skin); (2) mucosa-derived specimens (*n* = 14; palate, oral mucosa, “lip pits” and gingiva); (3) mucocutaneous junction area-derived specimens (*n* = 53; CLP lip, upper lip, lower lip and lip scar); (4) tooth-derived specimens (*n* = 13; PDL and dental pulp) (Figure 4A).

**TABLE 1:** Total status of our cell bank, including the tissue category, number of samples, cell types that have been successfully isolated, donor sex and age as well as disease status (specifics).

	Category	# Samples	Cells		Donor Sex			Donor Age	Specifics			
			Ep	Fb	M	F	n.a.		healthy	diseased	CFA	
											ns	s
**Skin**	**Forehead**	1	X	X	1	0	0	2–4 years	1			
	**Eyebrow**	1	X		0	0	1	2–4 years	1			
	**Ear**	1	X	X	0	0	1	n.a.	1			
	**Nose**	1	X	X	1	0	0	2–4 years	1			
	**Facial skin**	8	X	X	5	3	0	3–5 months			7	1× GH (F)
**Mucocutaneous junction area**	**CLP Lip**	48	X	X	30	18	0	3–5 months			46	1× VWS (M) 1× T13 (F)
	**Upper Lip**	3	X	X	1	1	1	3–5 years	3			0
	**Lower Lip**	1	X	X	1	0	0	19 years				1× VWS (M)
	**Scar (lip)**	1		X	1	0	0	19 years				1× VWS (M)
**Mucosa**	**Palate**	2		X	1	1	0	>18 years	2			
	**Oral Mucosa**	4	X	X	2	2	0	3–5 months			4	
	**“Lip Pits”**	1	X	X	1	0	0	19 years				1× VWS (M)
	**Gingiva**	7	X	X	4	2	1	>18 years	4	2		1× VWS (M)
**Teeth**	**Periodontal Ligament**	10		X	5	3	2	>18 years	10			
	**Dental Pulp**	3		X	2	1	0	>18 years	3			

*Diseased gingiva samples were received from periodontitis-affected patients. Ep: epithelial cells; Fb: fibroblasts; M: male; F: female; n.a.: not available; ns: non-syndromic; s: syndromic; CFA: craniofacial anomaly; GH: Goldenhar syndrome; VWS: Van der Woude syndrome; T13: Trisomy 13. Cells that have been used in this study, including cell name, donor sex, donor age, and characteristics are indicated.*

The majority of our biopsy collection (60/92–65%) is represented by discarded tissue excised during corrective surgeries of CFAs. In addition, we biobanked four pediatric healthy facial skin specimens (4/92–4.3%) and 3 healthy lip samples (3/92–3.3%) obtained during surgeries for minor injuries (i.e., lip laceration, accidental cuts). The remaining samples were collected as tissue remnants after routine dental treatments (21/92–22.8%) performed at the Dental School, University of Bern or after secondary surgeries at the Pediatric Surgery Division, Children’s Hospital, Bern (4/92–4.3%) (Figure 4B).

From this cohort of tissue biopsies, we so far processed 70 biopsies (70/92–76%) and were able to isolate cells from 69 samples (success rate 99%). The only tissue we were not able to obtain cellular outgrowths was a gingiva sample. We successfully isolated and purified 49 pairs of matching keratinocytes and fibroblasts (49/69–71%), while for 7 (7/69–10%) biopsies we could extract either only keratinocytes (eyebrow) or only fibroblasts (3 facial skin, 2 palate, and 1 oral mucosa biopsies). For additional 13 (13/69–19%) specimens (PDL, dental pulp, “lip pits” and scar) the tissue origin did not allow the isolation of keratinocytes. We did not yet perform cell extractions from 22 biopsies. Notably, we have remaining tissues of successful cell outgrowths for 48 samples (48/69–69%), which are stored in freezing medium in the liquid nitrogen tank for potential cell re-isolation in the future (Figure 4C).

Additionally, our biobank contains cells isolated from 10 more samples independent from the cranio-/orofacial region (Supplementary Table 3). This cohort resulted in eight successful matching keratinocyte/fibroblast pairs (seven foreskins, one thumb derived from polydactyly), while for two biopsies we only could get fibroblasts (synovia and ankle scar from a pigmented villonodular synovitis). The complete state of our biobank (CFA and other sources) is shown in Supplementary Figure 1.

### Our Cell Bank Offers Novel Tools and Models for Personalized Precision Medicine

#### Establishing Cell Models for the Study of Rare Human Conditions *in vitro*

In 5–7% of all CLP cases, orofacial clefting can be part of rare syndromes that present with additional anomalies outside of the region of clefting and that are usually caused by a single gene mutation, chromosomal abnormalities or exposure to teratogens during pregnancy (Mossey and Modell, 2012; Leslie and Marazita, 2013). In fact, at least 275 syndromes have been identified, which are associated with a clefting phenotype (Leslie and Marazita, 2013). As such, primary cells isolated from syndromic CLP-affected individuals are also very appealing for the establishment of clinically relevant cell models for the study of rare conditions, as exemplified below. Among our cohort, there are cells isolated from four individual patients affected by rare human diseases/syndromes, Van der Woude syndrome (VWS) (two patients) (Van Der Woude, 1954), Patau syndrome (Trisomy 13) (Miryounesi et al., 2016) and GH (Bogusiak et al., 2017). Short descriptions of the main features associated with these syndromes are reported in Figure 5 (Overview).

Regarding VWS, we have tissues and the corresponding cells from 2 affected patients: an infant (VWS1), who underwent primary corrective cleft lip surgery (CLP lip biopsy) and a 19 year old individual (VWS2), who had secondary surgery for functional as well as esthetical reasons (biopsies of the gingiva, lower lip, connective tissue of the lip pits, and the scar tissue from the primary corrective surgery to close the upper lip) (Figure 5A, Biopsies). While we were able to isolate both fibroblasts and keratinocytes from the lip and the gingiva tissues, we only managed to obtain fibroblasts from the scar and the “lip pits” connective tissue. We analyzed all cells for their morphologies as well as epithelial (E-Cadherin)- and mesenchymal-(FN)specific markers (Figure 5A, patient-derived cells). In addition, we prepared FFPE blocks and tissue sections from all four VWS2-derived biopsies. H&E and Toluidine Blue staining allow a preliminary descriptive appreciation of the tissue architecture, which could be further investigated by immunohistochemistry (IHC) (Figure 5A, Histology). By IHC staining of some sections, we were able to highlight the epidermis of the lip by E-Cadherin expression at adherent junctions between epithelial cells, scar tissue by strong positivity for α-smooth muscle actin (αSMA) in the stroma, and to confirm presence of high amounts of striated muscles in the “lip pit” tissue, which were already visible in the H&E sections, by Myosin fast II (MYO2) (Figure 5A, Histology).

Cellular and molecular analyses of the patient-derived cells clearly demonstrated proof-of-concept: the original hallmarks of the syndrome are retained in the cells *in vitro* and represent promising study tools. Using the isolated keratinocytes (VWS1) we were able to confirm and thoroughly characterize a novel *IRF6* variant (c961_965delGTGTAinsC), which has been initially sequence-identified by the Division of Human Genetics, Children’s Hospital Bern (Degen et al., 2020). Here, we show that in keratinocytes derived from the VWS2, both IRF6 protein as well as mRNA levels are comparable to VWS1, but robustly decreased when compared to non-syndromic (pathogenic variants in the known VWS-associated genes *IRF6*, *GRHL3*, and *NME1* have been excluded by whole exome sequencing) CLP- and healthy lip-derived keratinocytes. These data fit to the assumed notion that VWS-causing IRF6 mutations often result in haploinsufficiency of IRF6 (de Lima et al., 2009; Degen et al., 2020). Therefore, without knowledge of the underlying gene defect causing VWS in the second individual, we can speculate that the affected individual harbors a pathogenic IRF6 variant (Figure 5A, *In vitro* characteristics). In addition, gene expression analyses of the four different fibroblast cultures derived from the VWS2 individual allowed us to show that also fibroblasts retain their *in vivo* characteristics *in vitro*. Indeed, the scar-derived fibroblasts significantly present higher levels of the scar markers (Ehrlich et al., 1994; Tsou et al., 2000; Shinde et al., 2017) αSMA, *Collagen I* (*Col1*) (data not shown) and *Collagen III* (*Col3*) compared to the fibroblasts derived from other tissues (Figure 5A, *In vitro* characteristics), fitting to our IHC observations.

Among our cohort of tissue donors, there is also an individual affected by Trisomy 13 (Figure 5B), who underwent corrective surgery of the condition-associated orofacial cleft lip. Successful isolation of both keratinocytes and fibroblasts will allow scientists to study specific aspects of Trisomy 13 using clinically relevant cells (Figure 5B, Patient-derived cells). We collected the corresponding tissue as FFPE blocks. The initial H&E staining shows the complex anatomical structure of the lip as it represents a transition zone between mucosal and skin tissue (Figure 5B, Histology). In addition, we show presence of three chromosomes 13 by karyotype analysis (Figure 5B, *In vitro* characteristics).

Finally, we received a biopsy from a patient who presented with preauricular skin tags (Figure 5C, Overview and Biopsies), a typical feature of GH-affected individuals (Dole et al., 2017). From the biopsy, we were able to isolate and characterize patient-derived keratinocytes and fibroblasts (Figure 5C, Patient-derived cells). Such cells could represent an unprecedent research opportunity for scientists interested in this rare syndrome, since so far only one zebrafish model has been successful (Lopez et al., 2016). Next to the cells, we also biobanked FFPE blocks. Notably, H&E, Toluidine Blue and cartilage-specific Safranin-O staining confirmed the presence of pathological cartilage in these GH-associated skin tags (Figure 5C, Histology).

Our proof-of-concept study clearly shows that the patient-derived cells, often in combination with the corresponding FFPE blocks, represent promising tools to study rare human conditions and that the original cell/tissue characteristics are retained *in vitro*.

#### Potential Applications of Primary Cells in Preclinical and Translational Research

Finally, we would like to illustrate the translational potential of our living CFA cell bank in regard to regenerative and personalized medicine as well as to pharmaceutical, cosmetic, oral health and dental material industry.

##### Three-dimensional (3D) personalized skin models

Our approach of isolating keratinocytes as well as fibroblasts from the same tissue donor is very promising for the development of personalized 3D organotypic skin models, which mimic the *in vivo* skin as much as possible (Ravi et al., 2015). In Figure 6A we present preliminary data on the establishment of 3D-skin differentiation models (keratinocytes only) as well as on full thickness skin models (fibroblasts and keratinocytes) using non-syndromic CLP patient-derived cells. H&E stainings of both models indicate that both assays result in models that nicely resemble the *in vivo* skin with multiple superficial keratinocyte layers. Loricrin, a differentiation marker of the granular layer of epithelial cells can be detected in superficial keratinocyte layers (green), while PCNA (red) is only expressed in the basal proliferating keratinocytes (Figure 6A). Both observations are identical to the *in vivo* situation.

##### Modern dentistry and biomaterials

Our provision of cells isolated from most of the tissues represented in the oral cavity could be highly beneficial for dental material companies, in order to improve and advance the development of their products. For instance, primary gingival fibroblasts could represent an optimal model for studying the adhesion of soft tissues to the dental implant abutment or to dental composites for the treatment of submucosal lesion, while PDL and dental pulp fibroblasts may represent models for studying the efficacy of treatment for periodontal and dental regeneration, respectively. In addition, also the oral health industry (e.g., companies interested in the development of oral rinses or toothpastes) may benefit from clinically relevant cells available in our cell bank. Such cells could be used for testing the cytotoxic effects of new compounds in newly developed products. Using keratinocytes and fibroblasts isolated from healthy gingiva donors, we established straight-forward MTT assays to analyze the cytotoxic effects of commercially available oral rinses on paired gingiva-derived fibroblasts and keratinocytes (Figure 6B).

##### Tissue engineering and regenerative medicine

Our cohort of primary fibroblasts also shows certain criteria that have been attributed to MSCs: they are plastic-adherent and show a spindle-like morphology and express variable amounts of the MSC surface markers *CD73*, *CD90*, and *CD105*. Furthermore, our preliminary *in vitro* differentiation experiments revealed that similar to MSCs, PDL fibroblasts could be induced into bone forming cells as assessed by staining for Ca^2+^ deposits by Alizarin Red S (Figure 6C). These initial observations let us speculate that our patient-derived cells may represent a viable and easily accessible source of cells likely offering some regenerative potential. Clearly, further experimental evidence is warranted. However, we are encouraged to believe that the fibroblasts in our cell bank could be beneficial for the tissue donors themselves later in life if they might benefit from autologous, regenerative therapeutic options.

## Discussion

Discovery biomedical research is the foundation for an ever-increasing gain of knowledge concerning the biological processes underlying physiological as well as pathological craniofacial development. In the past years, significant progress has been made by using various model organisms to study genes required for proper face morphogenesis. More recently, this approach has been complemented by the availability of novel, state-of-the-art technologies allowing the identification of disease-causing gene mutations or chromosomal aberrations driving various anomalies. Thanks to these approaches, hundreds of genes and variants have been found to be linked to specific CFAs. However, scientific activities also envision to understand how certain gene mutations affect the behavior of cells and to establish genotype-phenotype correlations (Degen et al., 2020). Immortalized cell lines usually are used in such studies. However, nowadays, the scientific community is aware that often immortalized cells fail to recapitulate the *in vivo* situation as they often markedly differ in their phenotypes as well as genotypes from the original tissues. In addition, there are several reports about contaminated and misidentified cell lines (Masters, 2010; Lorsch et al., 2014), producing irreproducible results, on which many scientific discoveries and publications rely on. Therefore, there is urgent need for the availability of clinically relevant cell models that retain their original characteristics and that can be used as *in vitro* tools to further solidify the understanding of how genes affect cellular behavior in studying CFAs.

Our effort in establishing a cell biobank represents an unprecedented tool for the scientific community, who is interested in studying CFAs or periodontal/dental-related investigations. Such a project is only possible in a collaborative effort with the clinical counterparts, which provide all clinical samples. The availability of optimal cell models that retain their original characteristics (Figure 5) should help to advance discovery research on CFAs (see section “Establishing Cell Models for the Study of Rare Human Conditions *in vitro*”), but also could be of great benefit for the tissue donors themselves, who can rely on a personal repository of autologous cells at any moment of their life (Figure 6). In this regard, it is worth noting that in the literature cranio-/orofacial tissues have been described as very rich sources of progenitor cells or MSCs, with potential application for regenerative therapies (Sanz et al., 2015; Ebrahimi and Botelho, 2017). The International Society for Cellular Therapy (ISCT) defined a set of three minimal criteria that a cell has to fulfill in order to be viewed as an MSC (Dominici et al., 2006). However, the true identity of MSCs is still debatable as there is increasing evidence in the literature annotating similar characteristics to fibroblasts (Ichim et al., 2018; Soundararajan and Kannan, 2018). In this study, we show that some of the main characteristics defined for MSC are present also in our set of patient-derived fibroblasts (Figure 6). Clearly, more basic and functional studies are required to assess the clinical potential of patient-derived fibroblasts as alternatives to MSCs. However, our preliminary investigations let us believe that our cell bank might be beneficial for the tissue donors themselves for personalized regenerative therapies in their future.

To be successful and relevant for the economy and society, biomedical research increasingly relies on the existence and usage of data and samples stored in human biobanks (Hewitt, 2011; Coppola et al., 2019). Biobanks are organized collections of human biological samples and associated patient data, which are stored over lengthy periods allowing the availability of resources for research efforts. However, the establishment and the maintenance of human biobanks raises multiple ethical and juridical questions, which need to be addressed (Ashcroft and Macpherson, 2019). The acquisition, storage, and use of patient-derived specimens is indeed subject to stringent ethical as well as legal regulations. The protection of the privacy and identity of the research participants should be the top priority in all biobanking efforts. Unfortunately, there is not yet a harmonized legislative framework available within European countries regulating biobanking activities (Kaye et al., 2016). For the establishment of our CFA biobank, we included measures according to the https://www.wma.net/policies-post/wma-declaration-of-helsinki-ethical-principles-for-medical-research-involving-human-subjects/ and to the Swiss Human Act (HRA), which regulate the protection of the privacy and identity of participants on a global level and in Switzerland, respectively. First of all, all the projects involving the use of our cell models have to be approved by the regional research ethical committee. In addition to this, external investigators interested in using our cells are required to undersign a Material Transfer Agreement (MTA), a contract governing the transfer, intended use and rights of the research material. Secondly, all study participants or their legal representative in case of minors, are informed about the research and gave an informed consent, which allows individuals to decide whether and how their tissues samples can be used for research purposes. In both cases, patients have always the right to decide whether their personal data and tissue samples can or cannot be used in an encoded way for future biomedical research. Participants are also informed about the possibility to withdraw their consent at any time, which further exemplifies the protection and respect toward each individual. Notably, all the samples stored in our collection, are derived from planned and necessary surgical interventions, and all the biospecimens would have been otherwise discarded. Therefore, none of the participants experiences any supplemental consultation time, harm or any other disadvantages by donating their tissue for research purposes. Finally, we implemented various technological data security measures including the appropriate IT infrastructure in order to securely store patient data in a protected database.

In our workflow, the improvement of the protection of the patient’s privacy has been obtained by defining clear and strict responsibilities shared between the Children’s Hospital/Dental School and the research laboratory for the collection and encoding of the samples. All the clinically related data and work, such as the written consents, consultation and the diagnosis, are securely stored at the hospital/dental clinic. The laboratory receives the tissue sample in a pre-labeled tube (e.g., 001) in combination with minimal patient information (such as date of the surgery, age and gender) and the origin of the sample (e.g., bilateral cleft, gingiva). If applicable, the laboratory is also informed about the presence of any syndromes associated with the patient. This system does not allow the identification and tracking of patients and hence protects their privacy. If future research projects require more information about the tissue sample donor (e.g., patients requiring regenerative therapies using their own cells), this can only be achieved in consultation with the involved and trained personnel of the clinics. Clearly, it is our responsibility to regularly train all people involved in the biobanking process and to audit all our activities to ensure correct adherence to our guidelines.

We strongly believe that our approach in setting up a living cell repository for the cranio-/orofacial region derived from otherwise discarded tissues is unique and will represent an important asset for discovery and translational research on cranio-/orofacial anomalies as well as an essential tool to be combined with animal studies. 3D skin models established from patient-derived cells can be used as personalized organotypic cultures for drug screenings (e.g., to obtain better wound healing using CLP cells), cosmetics (facial skin cells), toxicity tests (oral cavity cells), bacterial infections related to periodontitis (gingiva cells) as well as for clinically relevant discovery research trying to elucidate specific molecular mechanisms. For instance, we applied such models in the understanding of how specific gene variants causing CLP and/or VWS affected the differentiation potential of skin and mucosa cells (Degen et al., 2018, 2020). Another research area that might benefit from such clinically relevant primary cells is dentistry. For the long-term success of regenerative procedures in dentistry, the integration of biomaterials within the oral cavity is of great clinical importance (van Steenberghe et al., 1991; Tayebi and Moharamzadeh, 2018). This largely depends on the behavior of cells at their interface and, particularly, on their initial attachment, adhesion and spreading (Anselme, 2000). However, these events can also be affected by the characteristics of the material itself, and this is the reason for why the development of more and more performant materials is currently a trend on the market. In spite of this, most of the available materials used in the daily clinical practices lack *in vitro* data, which could support and explain their clinical performance on a cellular level regulating biological events at the biomaterial interface (Parisi et al., 2020; Toffoli et al., 2020). Our primary dental-related cells could fill this gap and provide an optimal source for clinically relevant and tissue-specific cells. We are well aware that such a biobanking effort is time-consuming and requires a financial investment (e.g., infrastructure, IT support, trained personnel). Nevertheless, we are convinced that such a cell bank might be required in the pursuit of a complete understanding of specific CFAs, from which patients may benefit in the future.

In summary, here we presented our proof-of-concept study that patient-derived cells from the cranio-/orofacial region have great potential in discovery and translational research. We detailed our workflow and the main processes that we apply for the ongoing generation of our biobank to (1) promote, boost, and advance biomedical research on CFAs with high-quality primary cells, (2) have healthy primary control cells available from various cranio-/orofacial tissues, (3) establish patient-derived cell models for translational research such as drug testing using clinically relevant cells, (4) store patient-derived cells that could be used at a later time as an autologous source for personalized regenerative applications, and (5) gain more and urgently needed knowledge on the biological processes underlying various CFAs, which will be of great benefit to CFA-affected patients.

## Data Availability Statement

The datasets generated and/or analyzed during the current study are available from the corresponding author on reasonable request.

## Ethics Statement

This work was performed according to the Ethical Principles for Medical Research Involving Human Subjects as defined by the World Medical Association (WMA Declaration of Helsinki–Ethical principles for medical research involving human subjects. Available at: https://www.wma.net/policies-post/wma-declaration-of-helsinki-ethical-principles-for-medical-research-involving-human-subjects). Isolation of human cleft lip- as well as control tissue-derived cells and their analyses for this study had been approved by the Regional Research Ethic Board (Kantonale Ethikkommission of Bern, Switzerland, protocol number: 2017-01394). Informed written consent was obtained from the patient or their legal representatives.

## Author Contributions

LP, PK, EG, SR, and MD performed the experiments and analyzed the data. LP and MD wrote the manuscript. LP, MD, and CK initiated the project and planned, coordinated, and designed the experiments. DB was responsible for the histological analyses. MD and CK critically revised the manuscript and provided support throughout the project. IS and GL performed the clinical work (CLP and other CFAs). ASt and ASc performed the clinical work (dental-related biopsies). All authors critically reviewed the manuscript.

## Conflict of Interest

The authors declare that the research was conducted in the absence of any commercial or financial relationships that could be construed as a potential conflict of interest.

## Footnotes

1 https://pga.mgh.harvard.edu/primerbank/

2 www.ncbi.nlm.nih.gov/tools/primer-blast
