# Supplementary material for: A Living Cell Repository of the Cranio-/Orofacial Region to Advance Research and Promote Personalized Medicine
Source: Front Cell Dev Biol. 2021 Jun 10;9:682944. doi: 10.3389/fcell.2021.682944 (PMC8222786; doi:10.3389/fcell.2021.682944)
Supplement: Supplementary file 1 [file Data_Sheet_1.pdf]

Supplementary Material

A Living Cell Repository of the Cranio-/Orofacial Region to Advance Research and Promote Personalized Medicine

Ludovica Parisi[1], Patrick O Knapp[1], Eleftheria Girousi[1], Silvia Rihs[1], Giorgio C La Scala[2], Isabelle Schnyder[3], Alexandra Stähli[4], Anton Sculean[4], Dieter D Bosshardt[4,5], Christos Katsaros[1], Martin Degen[1]*

*** Correspondence:** Martin Degen
martin.degen@zmk.unibe.ch

1 Supplementary Tables

Gene	Forward (5' - 3')	Reverse (5' - 3')
FN	CCATTATTGGGTACCGCATCACA	AGGAGGAACAGCCGTTTGTTGT
KRT14	GGCCTGCTGAGATCAAAGACTAC	CACTGTGGCTGTGAGAATCTTGTT
CDH1	AGAACGCATTGCCACATACACT	TCTGATCGGTTACCGTGATCAA
TNC	TCAAAGACGTGCCAGGAGAC	TCTGTCTGGGAAACACGTCG
LAMA3	TCAGCACATATTATCTGGGAGG	AAATTTTTCATGCAGCCTCG
TGFß1	TCCTGTGACAGCAGGGATAAC	TCCGTGGAGCTGAAGCAATA
IRF6	GCTCTTCCATATCATGGCCCTC	CTACAGCCCAGGCCTTAAAAA
aSMA	CAGGGCTGTTTTCCCATCCAT	GCCATGTTCTATCGGGTACTTC
COL3	AACAGGAAGCTGTTGAAGGAGG	CTGTGGGCAAACTGCACAAC
CD73	CCAGTACCAGGGCACTATCTG	TGGCTCGATCAGTCCTTCCA
CD90	ATCGCTCTCCTGCTAACAGTC	CTCGTACTGGATGGGTGAACT
CD105	TGCACTTGGCCTACAATTCCA	AGCTGCCCACTCAAGGATCT
GAPDH	CTCTGACTTCAACAGCGACACCC	TCCTCTTGTGCTCTTGCTGGGGC

Supplementary Table 1. Sequence of the qPCR primers used in this study.

Assay	Protein	Type	Clone	Company
IF	Laminin-y2	monoclonal	E-6	sc-28330, Santa Cruz
	Fibronectin	polyclonal	/	Wehrle-Haller et al., 1991
	E-Cadherin	polyclonal	/	2084-1-AP, Proteintech
	Vinculin	monoclonal	hVIN-1	V9131, Sigma-Aldrich
WB	Fibronectin	polyclonal	/	Wehrle-Haller et al., 1991
	E-Cadherin	polyclonal	/	2084-1-AP, Proteintech
	IRF6	monoclonal	14B2C16	674502, BioLegend
	Vinculin	monoclonal	hVIN-1	V9131, Sigma-Aldrich
	ß-Actin	monoclonal	C4	sc47778, Santa Cruz
IHC	E-Cadherin	monoclonal	HECD-1	13-17000, Thermo Fisher Scientific
	Vimentin	monoclonal	3B4	M7020, Dako Agilent
	Alpha smooth actin	monoclonal	1A4	1A4, Sigma-Aldrich
	Skeletal Myosin, fast, Myo II	monoclonal	MY-32	M-4276, Sigma-Aldrich
	PCNA	polyclonal	/	ab18197, abcam
	Loricirn	polyclonal	/	PA5-30583, Thermo Fisher Scientific

Supplementary Table 2. Antibodies used in this study. IF: Immunofluorescence; WB: Western Blot; IHC: Immunohistochemistry.

Wehrle-Haller, B., Koch, M., Baumgartner, S., Spring, J., and Chiquet, M. (1991). Nerve-dependent and -independent tenascin expression in the developing chick limb bud. *Development* 112**,** 627-637.

	Category	# Samples	Cells		Donor Sex		Donor Age	Specifics	
			Ep	Fb	M	F		healthy	diseased
Skin	Thumb	1	X	X	0	1	2-4 years		1
	Scar (ankle)	1		X	0	1	39 years		1
MJ	Foreskin	7	X	X	7	0	4-8 years	7	
M	Synovial Membrane	1		X	0	1	39 years		1

Supplementary Table 3. Non-cranio-/orofacial-derived cells. MJ: mucocutaneous junction area; M: mucosa; Ep: epithelial cells; Fb: fibroblasts; M: male; F: female.

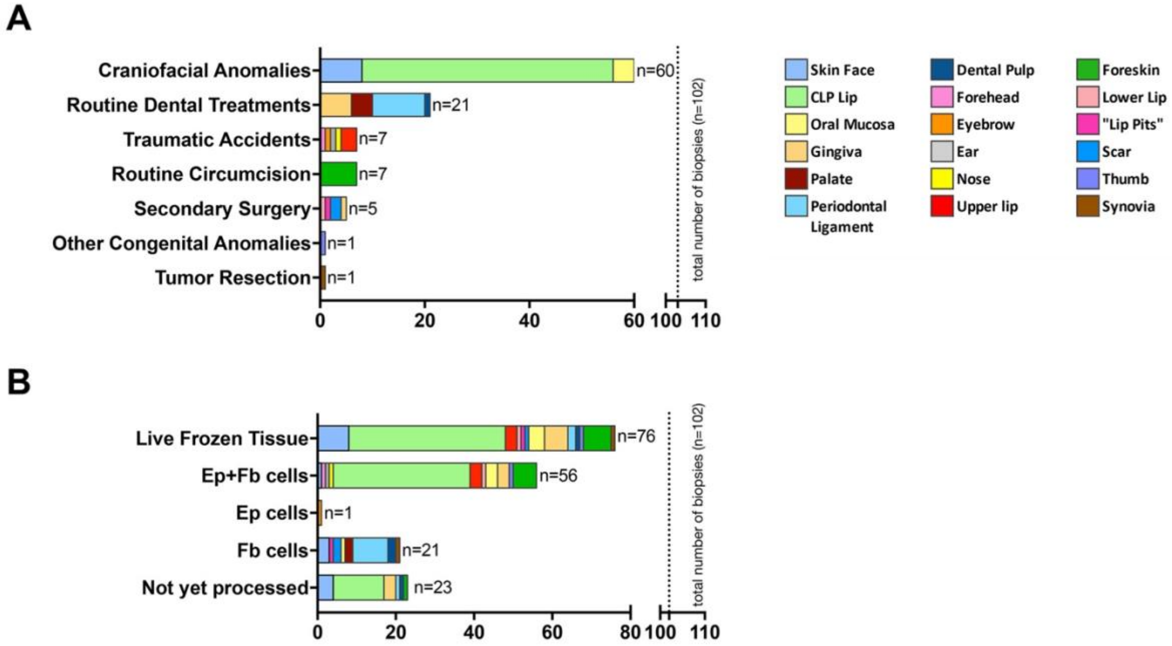

Supplementary Figure 1: Overall description of our living cell repository. (**A**) Bar chart reporting the source of the specimens that were obtained for the generation of the living cell biobank. Total number of biopsies: 102 (92 biopsies derived from the cranio-/orofacial region, 10 biopsies derived from other regions). (**B**) Bar chart of the outcome after tissue biopsy processing. Total number of biopsies: 102. The color code is shown in the legend.
